# Impact of Economic Factor, Percent Vaccination, Healthcare Quality, and Population Density on Coronavirus Disease 2019 (COVID-19) Mortality Rates: A Global Analysis in 2023

**DOI:** 10.7759/cureus.80582

**Published:** 2025-03-14

**Authors:** Sitthichai Kanokudom, Natchanid Piamsa-nga, Kantachai Ratanapanich, Kritpaul Prasattongosoth, Monile Suchitbharabitya, Punpiti Piamsa-nga, Natthinee Sudhinaraset, Sittisak Honsawek, Yong Poovorawan

**Affiliations:** 1 Center of Excellence in Clinical Virology, Faculty of Medicine, Chulalongkorn University, Bangkok, THA; 2 Department of Computer Engineering, Faculty of Engineering, Kasetsart University, Bangkok, THA; 3 Center of Excellence in Osteoarthritis and Musculoskeleton, Faculty of Medicine, Chulalongkorn University, Bangkok, THA

**Keywords:** coronavirus disease 2019 (covid-19), gross domestic product (gdp) per capita, infant mortality rate (imr), mortality rate, percent vaccination, population density, relationship

## Abstract

The coronavirus disease 2019(COVID-19) pandemic, caused by the novel severe acute respiratory syndrome coronavirus 2(SARS-CoV-2), emerged as a global health crisis in late 2019, triggering unprecedented government actions worldwide. COVID-19 mortality varied based on many factors, with this study focusing on socioeconomic status and healthcare quality across 95 countries. Countries were classified by gross national income (GNI) per capita into high-income (HI), upper-middle-income (UMI), lower-middle-income (LMI), and low-income (LI) countries. Data were collected from databases in 2023, including gross domestic product (GDP) per capita, percent vaccination, infant mortality rate (IMR), and population density. The findings revealed that HI and UMI countries experienced significantly higher mortality rates compared to LMI and LI groups. Economic status, measured by GDP per capita, demonstrated a strong negative correlation with mortality in HI countries (Spearman's *r*=-0.562; *p*<0.001) but a strong positive correlation in LMI countries (Spearman's *r*=0.629; *p*=0.002). Vaccination coverage showed a significant strong negative correlation with mortality in HI countries (Spearman's *r*=-0.551; *p*<0.001), underscoring the importance of widespread vaccination in reducing mortality. IMR exhibited a very strong negative correlation with mortality in LMI countries (Spearman's *r*=-0.704; *p*<0.001), an unexpected finding warranting further investigation. No such correlation was observed in other groups. Population density showed no significant impact on mortality rates across all income groups (*p*≥0.05). These results highlight the importance of economic resources and vaccination efforts in reducing COVID-19 mortality, while population density showed no impact. Our research provides insights to guide future public health responses.

## Introduction

In late 2019, the world encountered an unprecedented health crisis with the emergence of a novel severe acute respiratory syndrome coronavirus 2 (SARS-CoV-2), the cause of the coronavirus disease 2019 (COVID-19). What initially appeared as a cluster of mysterious pneumonia cases in Wuhan, Hubei, China, quickly escalated into a global pandemic of historical magnitude. The extensive globalization networks of our modern society enabled the virus to cross borders rapidly, spreading to every continent by early 2020 [1]. This rapid transmission disrupted daily life, overwhelmed healthcare systems, and created widespread uncertainty. This prompted the World Health Organization (WHO) to declare a public health emergency on January 30, 2020, and officially recognized the outbreak as a pandemic on March 11, 2020.

The impact of COVID-19 has been multifaceted, affecting health, society, and the global economy. The health crisis resulted in millions of illnesses, hospitalizations, and deaths, leading to long-term complications for many patients and placing immense strain on healthcare systems [2]. Social and economic disruptions followed, with lockdowns affecting businesses, travel, and education, while social isolation exacerbated mental health issues. By late 2022, the development and distribution of vaccines, along with an increased understanding of the virus, enabled more effective management of the outbreak.

The initial outbreak of COVID-19 in Hubei, China, had an overall mortality rate of 2.3% among 44,672 confirmed cases as of February 11, 2020. Most deaths occurred in individuals aged 70 and older, with no reported deaths among those aged nine and younger [3]. Strict lockdown measures played a critical role in controlling the spread. However, as the virus spread globally, mortality rates varied widely. For instance, in the United Kingdom, the mortality rate increased from 6.7% to 7.2% by mid to late 2020 [1], while in European countries, rates ranged between 5.7% and 11.6% by the end of 2021 [4].

Vaccines were seen as a beacon of hope in ending the pandemic. By the beginning of 2021, the first COVID-19 vaccine, known as CoronaVac (Sinovac Biotech Co., Ltd., Beijing, China), received emergency use approval from the WHO, followed by AstraZeneca (University of Oxford/AstraZeneca, Oxford, UK), Pfizer (Pfizer-BioNTech Inc., New York City, NY, USA), Moderna (Moderna Inc., Cambridge, MA, USA), and others. These vaccines were in very high demand in many countries worldwide. Despite extensive use, these vaccines were unable to control the rapid spread of the virus globally. COVID-19 vaccination rates show significant disparities between countries [5], which could be influenced by vaccine hesitancy. Wealthier nations typically have greater resource availability and more efficient management, giving them better access to vaccines. Additionally, their well-developed infrastructure enables them to carry out rapid vaccination campaigns. Some countries face social and cultural barriers that impact vaccination rates [6]. Recently, multiple boosters of the existing vaccines have still been unable to prevent infection because the virus has a short incubation period and can mutate rapidly. This results in the virus evading the existing immunity or vaccine-induced immunity [7]. During the initial outbreak, the mortality rates for the Alpha and Delta waves were 2.62% and 2.01%, respectively. However, the mortality rate dropped significantly to 0.7% during the Omicron period [8].

Nevertheless, COVID-19 mortality rates are influenced by several factors such as economic status, vaccination coverage, population density, healthcare quality, and the constantly changing virus variants. Countries with higher economic status, measured by their gross domestic product (GDP) and gross national income (GNI) per capita, generally had better access to a wider range of healthcare resources and technologies, leading to improved healthcare services and outcomes. This also often resulted in higher life expectancy and lower infant mortality rates (IMR) [9], whereas countries with a lower economic status faced different challenges during the pandemic. The previous study demonstrated that higher vaccination rates, including booster doses, were associated with lower case hospitalization rates and fatality [10]. Similarly, other cohort studies conducted in Singapore and the United States showed that booster doses reduced the severity of COVID-19 infections during the Omicron wave [11,12]. Populations with elderly and those with underlying health conditions experienced higher mortality rates compared to the younger population [13]. Population density can exacerbate transmission, impacting mortality through increased exposure and potential strain on healthcare systems. High case numbers can overwhelm resources, leading to poorer outcomes. Effective public health strategies targeting these key areas are vital to reducing mortality and effectively managing infectious disease outbreaks. In some underdeveloped and developing countries with inadequate health and sanitation systems, symptoms of COVID-19 can be misdiagnosed, leading to improper treatment [14] and underreporting of the mortality rate.

This study aims to explore the relationship between COVID-19 mortality rates and various socioeconomic and healthcare factors across 95 countries in 2023. By analyzing data on economic status (referred to as GDP and GNI per capita), vaccine coverage (referred to as percent vaccination), healthcare system (referred to as IMR), and population density, we seek to generate insights into how these factors influenced COVID-19 mortality rates. Understanding these relationships will contribute to better preparedness and response strategies for future public health emergencies.

## Materials and methods

Study design and data collection

This cross-sectional study data was gathered from reliable databases related to COVID-19 for analysis in 2023. The study collected data from 95 out of 195 countries worldwide (approximately 50%), spanning Europe (n=25), Asia and Oceania (n=25), Africa (n=24), and North America and South America (n=21). The country names and their corresponding three-letter International Organization for Standardization (ISO) codes are provided in Appendix 1. This included comprehensive datasets across 10 parameters as follows: (1) population (n) in 2023; (2) population density (persons/km²) in 2023; (3) cumulative COVID-19 confirmed death cases (n) as of December 29, 2023; (4) cumulative COVID-19 confirmed death cases/100k population (ratio) in 2023; (5) percent vaccination (last updated March 13, 2023); (6) GDP (current USD) in 2023; (7) GDP per capita (current USD) in 2023; (8) GNI per capita, Atlas method (current USD) in 2023; (9) GNI per capita, purchasing power parity (PPP) (current international USD) in 2023; and (10) IMR (deaths/1,000 live births) in 2023.

Countries lacking available or updated data were excluded. This study does not involve direct research with human subjects and solely relies on publicly available data and aggregated information. Therefore, this study also is exempt from the requirement of obtaining ethical approval for human research and informed consent from participants.

Population and population density (persons/km²) data were gathered from Worldometer. The information was accessed by navigating to "Population by Country" and selecting the respective data for each country in 2023 [15]. GDP (current USD), GDP per capita (current USD), GNI per capita (current USD), and GNI per capita (PPP, current international USD) in 2023 were sourced from the World Bank using the relevant keywords above [16]. The percentage of COVID-19 vaccinations was sourced from The New York Times in 2023 [17]. The cumulative number of COVID-19 confirmed death cases was gathered through Our World in Data [18], with the data selected as of December 29, 2023. Furthermore, the cumulative number of death cases was divided by the population of each nation to calculate the death cases per 100k population. The IMR was collected through the Central Intelligence Agency (CIA) website in 2023 [19]. All information is shown in Appendix 1 and Appendix 2.

The study included 95 selected countries worldwide with complete data, providing a broad representation of global variations in factors potentially associated with COVID-19 confirmed mortality rates. First, we categorized the countries by income levels into four groups as suggested by the World Bank using the GNI per capita, Atlas method (current USD) in 2023 [20]. Countries are classified by GNI per capita as follows: high-income (HI): >13,845 USD, upper-middle-income (UMI): 4,466-13,845 USD, lower-middle-income (LMI): 1,136-4,465 USD, and low-income (LI): ≤1,135 USD. An exception was made for Afghanistan (AFG) and Qatar (QAT) having a cut-off based on 2022 (Appendix 1). The cumulative COVID-19 confirmed death case/100k population is referred to as a mortality rate. Economic status is indicated by GDP and GNI per capita. Healthcare systems are measured through the IMR, which refers to the number of infant deaths (<1 year) per 1,000 live births. The population density is measured in residents per square kilometer (persons/km^2^). We evaluated the relationship between the COVID-19 mortality rate and associated factors including GDP per capita, percent vaccination, IMR, and population density.

Data and statistical analysis

The data for death cases/100k population (mortality rate) and associated factors, including GDP per capita, percent vaccination, IMR, and population density, were presented as mean±standard deviation (SD). Statistical comparisons between groups across each factor were computed using the Kruskal-Wallis test in GraphPad Prism 10.2.1 (Dotmatics, Boston, Massachusetts, United States). Compact letter displays are calculated to assign letters to groups, where groups sharing the same letter are not significantly different, while those with different letters are.

Scatter plots and linear regression analyses illustrated the relationship between the COVID-19 mortality rate and associated factors. Correlation efficiency (Spearman's *r*) and statistical analyses of correlations were also conducted using GraphPad Prism 10.2.1. We performed an XY-plot between the mortality rate and each associated factor and then selected "Analyze" > "Multiple Variable Analyses" > "Correlation Matrix" > "No. Compute non-parametric Spearman correlation" > "OK" in Prism. Spearman's *r* was interpreted as follows: 0.00-0.19 (no or negligible), 0.20-0.29 (weak positive), 0.3-0.39 (moderate positive), 0.4-0.69 (strong positive), and ≥0.7 (very strong positive). Minus Spearman's *r* values indicated a negative correlation. A *p*-value of <0.05 was considered statistically significant.

## Results

Higher COVID-19 confirmed mortality rate in HI and UMI countries compared to LMI and LI groups

The mortality rate per 100k population was analyzed across HI, UMI, LMI, and LI countries. A total of 38 HI countries were included, with a mean mortality rate of 233.0±138.7 ratio (Appendix 3). The mean mortality rates for UMI, LMI, and LI countries were 168.9±141.3, 50.1±69.1, and 6.3±5.5 ratio, respectively. Mortality rates in HI and UMI countries were considerably higher compared to LMI and LI countries. Overall, the mean mortality rate across all countries was 149.9±145.4 ratio.

A strong negative correlation between the COVID-19 confirmed mortality rate and GDP per capita in HI countries but a strong positive correlation in LMI countries

**Figure 1:**
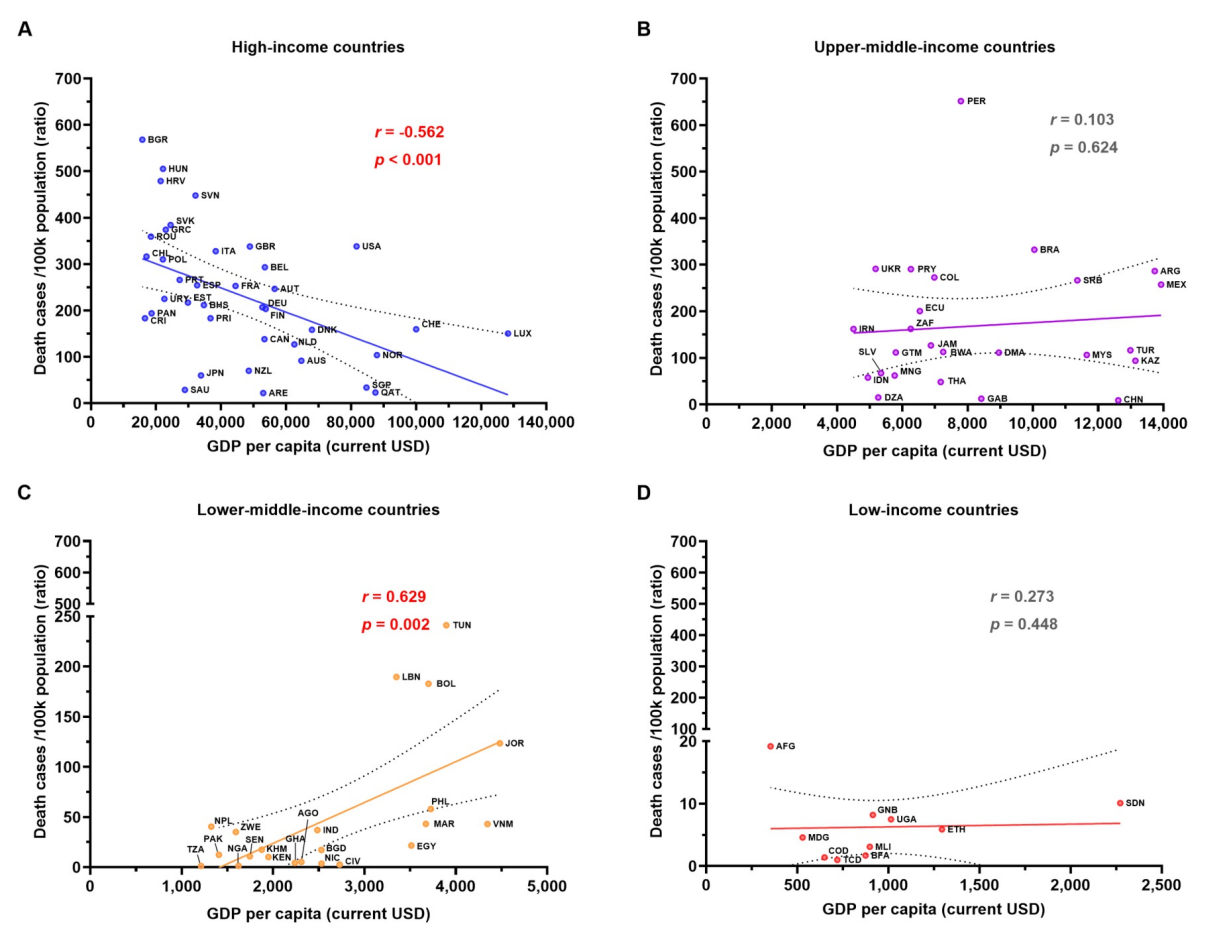
The correlation between the COVID-19 mortality rate and the GDP per capita in 2023 Panel (A) presents the data for HI countries (n=38), while panels (B), (C), and (D) show the data for UMI (n=25), LMI (n=22), and LI (n=10) countries, respectively. The X-axis represents GDP per capita in current USD, and the Y-axis represents the mortality rate (death cases per 100k population). The trendline indicates the relationship between the mortality rate and the GDP per capita. Spearman's *r* and statistical analyses of correlations are reported with a *p*-value of <0.05 considered statistically significant. COVID-19: coronavirus disease 2019; GDP: gross domestic product; HI: high-income; UMI: upper-middle-income; LMI: lower-middle-income; LI: low-income

The mean GDP per capita for 38 HI countries was 45,971.0±26,778.0 USD. Among this group, Luxemburg (LUX), Switzerland (CHE), and Norway (NOR) ranked as the top three income GPD per capita, respectively (Figure 1A). In contrast, the mean GDP per capita for 25 UMI, 22 LMI, and 10 LI countries was 8,439.0±3,116.0 USD, 2,647±1,027 USD, and 951.6±533.3 USD, respectively (Figure 1B-1D). For all countries combined (n=95), the mean GDP per capita was 21,299.0±26,479.0 USD (Appendix 3). GDP per capita was significantly lower in LI countries compared to the other three groups (Appendix 3).

A significant strong negative correlation was found between the mortality rate and GDP per capita in HI countries (Spearman's *r*=-0.562;*p*<0.001) (Figure 1A). This trend suggests that higher GDP per capita is associated with lower mortality in these HI nations. Conversely, no significant negligible positive correlation was observed between the mortality rate and GDP per capita in the UMI and LI groups, indicating no clear trend in these categories (Figure 1B, 1D). Interestingly, a strong positive correlation between the mortality rate and GDP per capita was observed in LMI countries (Spearman's *r*=0.629; *p*=0.002) (Figure 1C). This finding suggests that higher GDP per capita is strongly associated with lower mortality in HI countries. In contrast, the strong positive correlation in LMI countries may indicate an unusual trend or the influence of underlying factors linking GDP per capita and mortality, warranting further investigation.

A strong negative correlation between the COVID-19 confirmed mortality rate and percent vaccination in HI countries

**Figure 2:**
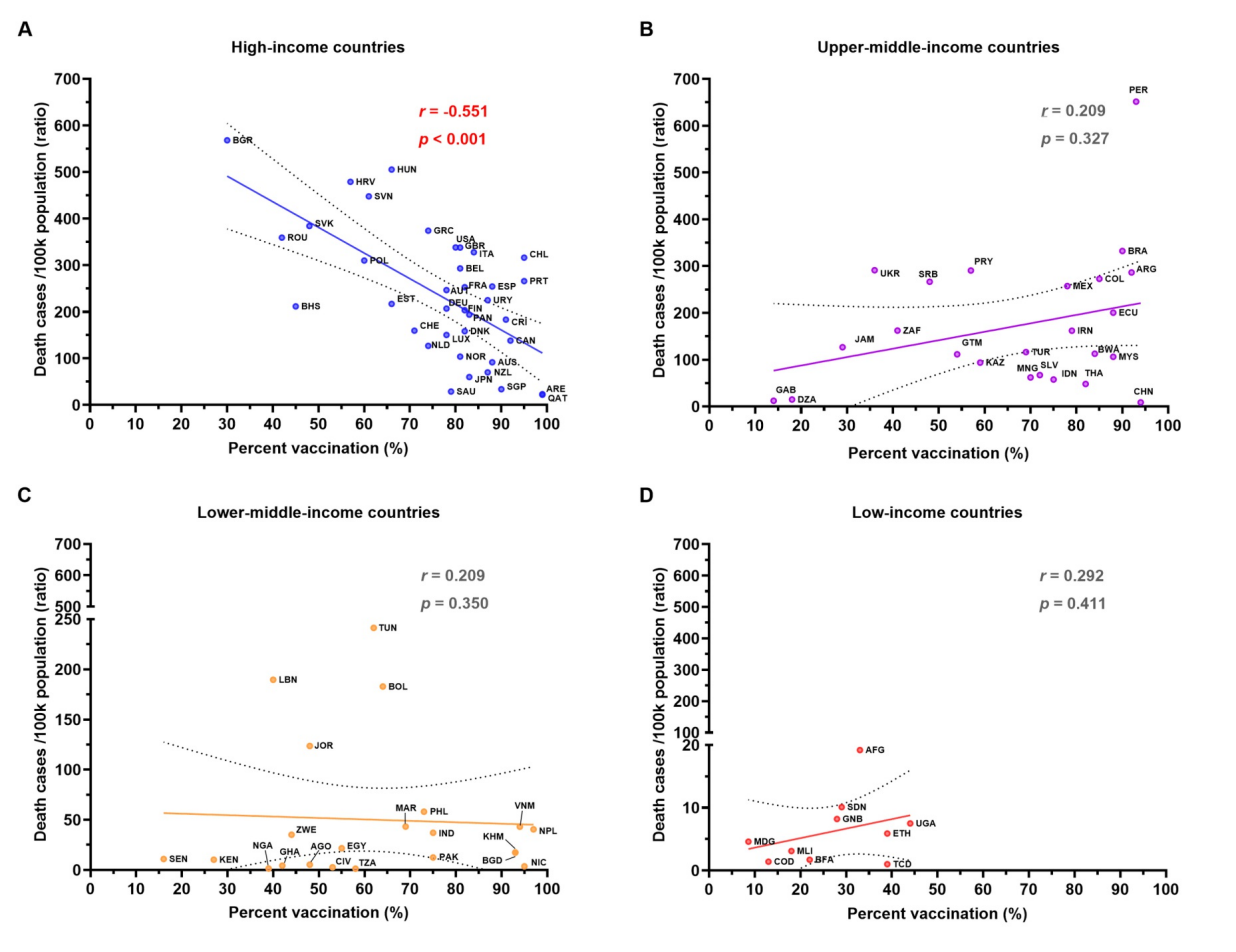
The correlation between the COVID-19 mortality rate and the percent vaccination in 2023 Panel (A) presents the data for HI countries (n=38), while panels (B), (C), and (D) show the data for UMI (n=25), LMI (n=22), and LI (n=10) countries, respectively. The X-axis represents the percent vaccination, and the Y-axis represents the mortality rate (deaths per 100k population). The trendline indicates the relationship between the mortality rate and the percent vaccination. Spearman's *r* and statistical analyses of correlations are reported with a *p*-value of <0.05 considered statistically significant. COVID-19: coronavirus disease 2019; HI: high-income; UMI: upper-middle-income; LMI: lower-middle-income; LI: low-income

In this analysis, Puerto Rico (PRI) from the HI group and Dominica (DMA) from the UMI group were excluded due to the absence of updated COVID-19 vaccination percentage data as of March 13, 2023 [17]. Among HI countries, Qatar (QAT) and the United Arab Emirates (UAE) had the highest vaccination rates, with over 99% of their populations receiving at least one dose (Figure 2A and Appendix 1). The mean percent vaccination for HI countries was 76.7±16.2%. In UMI, LMI, and LI countries, the mean percent vaccination was 66.5±24.3%, 61.8±23.3%, and 27.4±11.8%, respectively (Figure 2B-2D). A significantly lower percent vaccination was observed in LI countries compared to HI, UMI, and LMI countries (Appendix 3). The study showed that less than half of the population in LI countries received at least one dose of the vaccine. Overall, the mean vaccination rate across all countries was 65.2±24.5%.

Additionally, we conducted correlation analyses between mortality and percent vaccination. A statistically significant strong negative correlation between mortality rate and percent vaccination was found in HI countries (Spearman's *r*=-0.551; *p*<0.001) (Figure 2A), while no significant correlation between mortality rate and percent vaccination was found in the other three groups (Figure 2B-2D). This suggests that in HI countries, higher vaccination rates are strongly associated with a decline in mortality rates. The results also suggest that the percent vaccination is not associated with mortality rates in UMI, LMI, and LI countries.

A very strong negative correlation between the COVID-19 confirmed mortality rate and IMR in LMI countries, but no correlation in other groups

**Figure 3:**
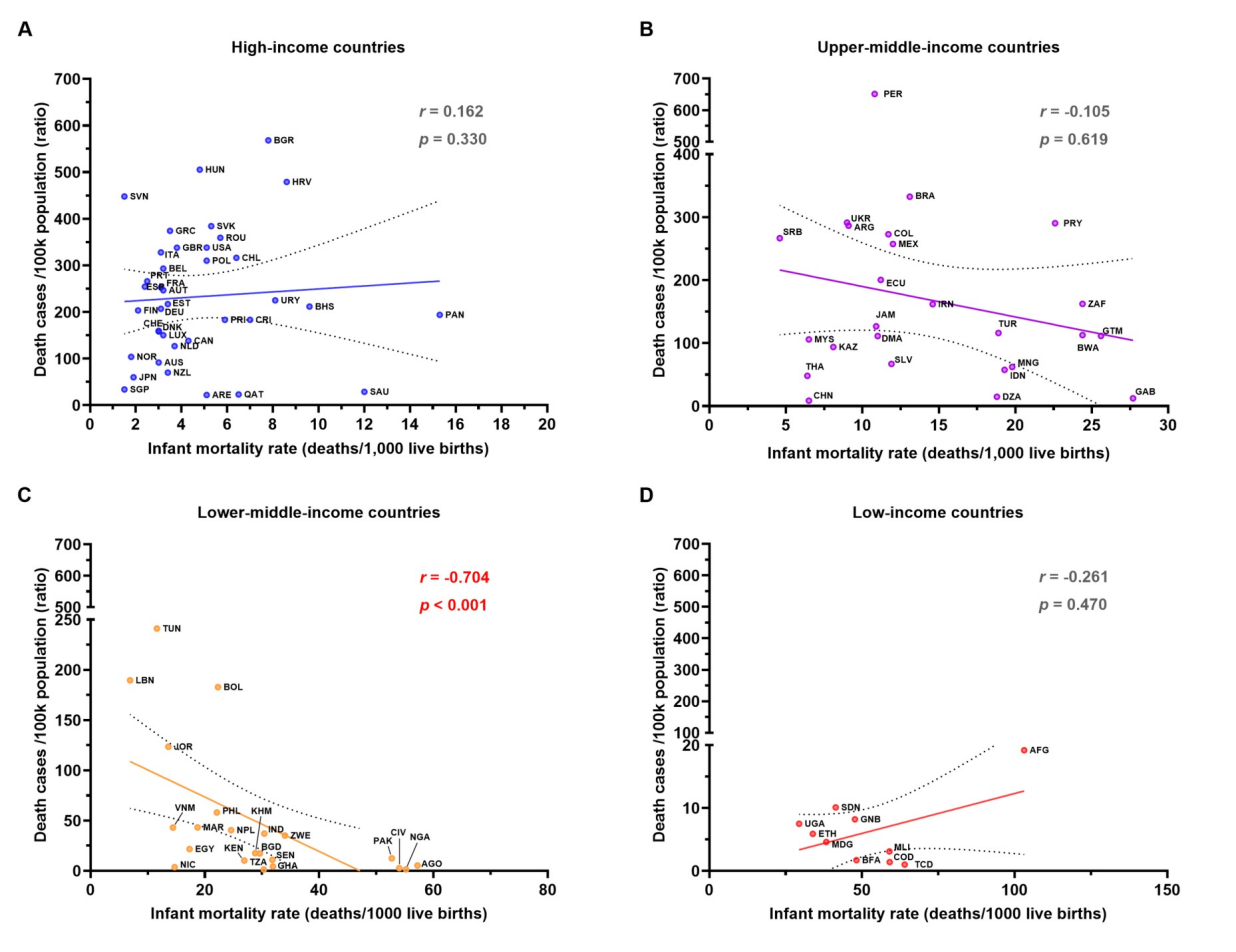
The correlation between the COVID-19 mortality rate and the infant mortality rate in 2023 Panel (A) presents the data for HI countries (n=38), while panels (B), (C), and (D) show the data for UMI (n=25), LMI (n=22), and LI (n=10) countries, respectively. The X-axis represents the infant mortality rate in deaths/1,000 live births, and the Y-axis represents the mortality rate (deaths per 100k population). The trendline indicates the relationship between the mortality rate and the infant mortality rate. Spearman's *r* and statistical analyses of correlations are reported with a *p*-value of <0.05 considered statistically significant. COVID-19: coronavirus disease 2019; HI: high-income; UMI: upper-middle-income; LMI: lower-middle-income; LI: low-income

Among the 38 HI countries, the mean IMR was 4.8±3.0 deaths/1,000 live births. The mean IMR was continuously increased to 14.4±6.8, 28.6±14.7, and 52.4±21.2 deaths/1,000 live births for the 25 UMI, 22 LMI, and 10 LI countries, respectively. Afghanistan (AFG) recorded the highest IMR at 103.5 deaths/1,000 live births. A comparison between groups revealed no significant difference in IMR between HI and UMI countries or between UMI and LMI countries. However, the IMR in LI countries was significantly lower than in the other three wealthier groups (Figure 3 and Appendix 3). The mean IMR across all countries was 17.8±19.2 deaths/1,000 live births.

The results showed a non-significant and negligible correlation between COVID-19 mortality rates and IMR among HI countries (Spearman's *r*=0.162; *p*=0.330) (Figure 3A). Similar to HI countries, no significant correlations were observed among the UMI and LI countries (Figure 3B, 3D). The finding indicates that IMR had no impact on the mortality rate in these countries. Unfortunately, a very strong negative correlation with statistical significance between mortality rate and IMR was found in LMI countries (Spearman's *r*=-0.704; *p*<0.001) (Figure 3C). This unexpected finding warrants further investigation to understand the hidden agendas driving this trend in LMI countries.

No correlation between the COVID-19 confirmed mortality rate and population density in all groups

**Figure 4:**
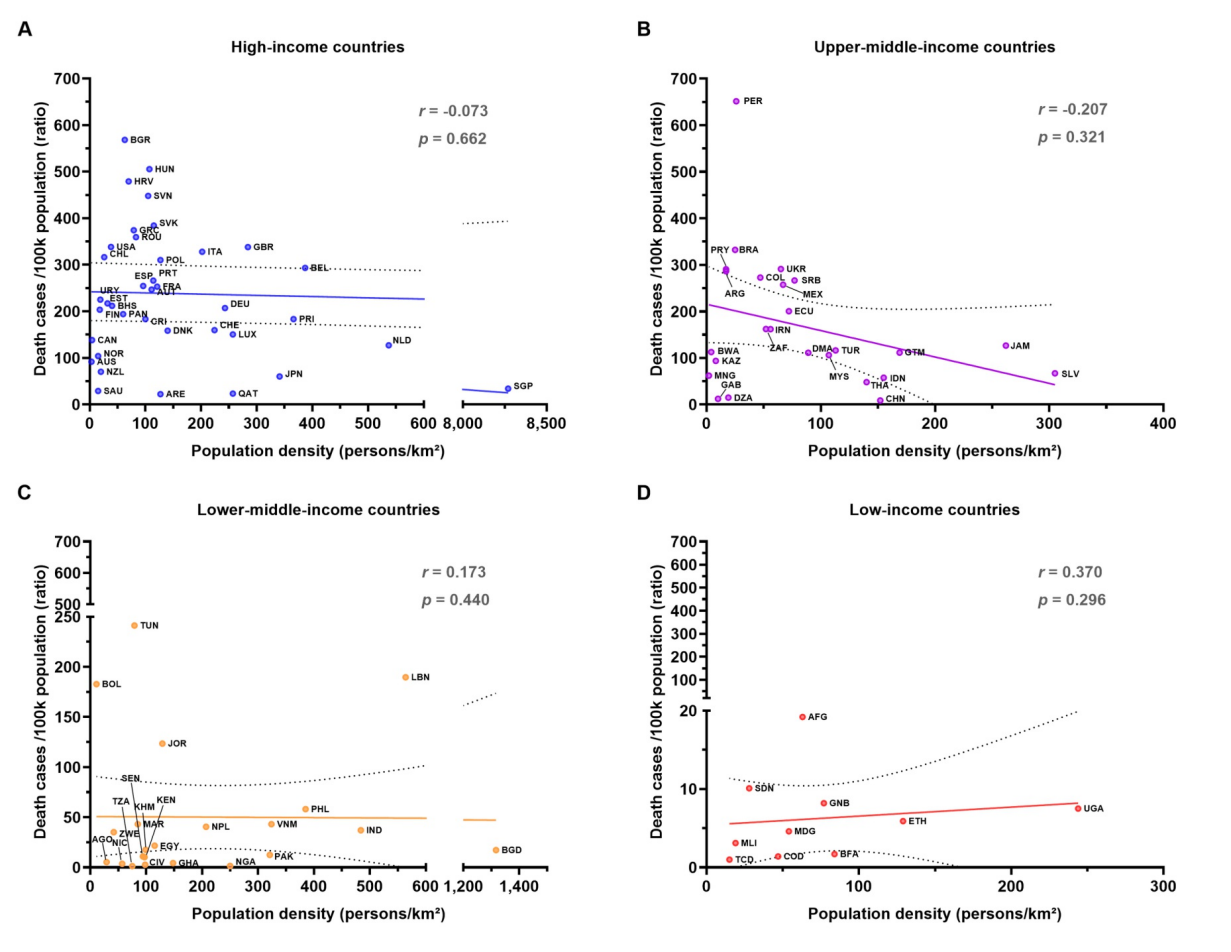
The correlation between the COVID-19 mortality rate and the population density in 2023 Panel (A) presents the data for HI countries (n=38), while panels (B), (C), and (D) show the data for UMI (n=25), LMI (n=22), and LI (n=10) countries, respectively. The X-axis represents the population density in persons/km^2^, and the Y-axis represents the mortality rate (deaths per 100k population). The trendline indicates the relationship between the mortality rate and the population density. Spearman's *r* and statistical analyses of correlations are reported with a *p*-value of <0.05 considered statistically significant. COVID-19: coronavirus disease 2019; HI: high-income; UMI: upper-middle-income; LMI: lower-middle-income; LI: low-income

The mean population density was 347.8±1,326.0, 82.2±79.1, 227.7±286.4, and 76.0±68.2 for HI, UMI, LMI, and LI countries, respectively. No significant differences in mean population density were observed between HI or LI countries and middle-income countries (Appendix 3). The most crowded country was Singapore (SGP), classified as an HI country, with a population density of 8,270.0 persons/km^2^. There were no significant correlations between mortality rate and population density across any group (*p*≥0.05), suggesting that population density had no discernible impact on COVID-19 mortality (Figure 4). 

## Discussion

The current study gathered a cumulative number of COVID-19 confirmed deaths per 100k population (mortality rate) during the pandemic from 2020 to 2023 across 95 out of 195 countries (approximately 50%). This study further investigated external factors contributing to the mortality rate associated with COVID-19 across countries classified by income levels. A significant negative correlation between GDP per capita and mortality rate was observed in HI countries, indicating that higher economic capacity may enable better healthcare access, resource mobilization, and pandemic response, ultimately lowering mortality. Conversely, a strong positive correlation in LMI countries suggests an unusual trend. This may be influenced by economic disparities within the group, with wealthier nations experiencing worse outcomes possibly due to higher urbanization, healthcare system strain, or the impact of comorbidities associated with transitioning economies. This phenomenon was supported by a previous study indicating that HI countries have demonstrated a greater capacity to battle the second and third waves of COVID-19 resulting in lower mortality, while the mortality rate in UMI and LMI countries has even increased [21]. Discrepancies in the correlation between GDP per capita and mortality were observed between HI and LMI countries. In HI countries, better healthcare infrastructure and greater access to medical resources contribute to lower fatality rates. In contrast, LMI countries may be affected by confounding factors such as underreporting due to limited testing and health surveillance. These findings suggest that GDP per capita alone does not fully capture a country's ability to mitigate COVID-19 mortality. Further research incorporating socioeconomic indicators like healthcare expenditure per capita and the Human Development Index (HDI) could provide deeper insights into these contrasting trends.

The findings revealed that HI countries demonstrated strong economic status as well as extensive vaccine coverage, measured by the percentage of vaccinated individuals. These factors significantly contributed to reducing COVID-19 mortality rates. Our research highlighted the impact of large-scale vaccination campaigns in improving health outcomes, particularly in wealthier (HI) countries. Our finding showed a statistically significant decrease in COVID-19 mortality rates as percent vaccination increased. This aligned with other studies showing that vaccines have been proven effective in preventing symptomatic infection and severe disease including hospitalization and death from COVID-19 [22-24]. However, no significant correlations were observed in UMI, LMI, and LI groups, suggesting that factors beyond vaccination rates, such as vaccine accessibility, equitable distribution, and healthcare delivery systems, may influence mortality in these regions. The low vaccination rates in LI countries, where less than half of the population received at least one dose, remain a critical concern. Moreover, this finding may align with a previous study conducted in 38 European countries, all of which were UMI or HI. The study showed a downward trend in new COVID-19 cases associated with an increase in GDP per capita [25]. 

Previous studies showed that IMR was used as a complex indicator to assess the impact of healthcare quality including health expenditure and outcome [9,26,27].Consequently, healthcare systems with low IMR are generally better equipped to manage pandemics [27], potentially contributing to lower COVID-19 mortality rates. However, this study found that IMR appeared to have no effect on mortality in HI, UMI, and LI countries. Interestingly, a very strong negative correlation in LMI countries suggests an unexpected link between IMR and COVID-19 outcomes. This could reflect underlying structural factors or data inconsistencies within LMI nations. Further research is required to explore the role of healthcare quality and access in influencing pandemic outcomes in these settings. 

Furthermore, no association between population density and COVID-19 mortality across any income group was observed. While densely populated countries like Singapore (SGP) had high population densities, their effective public health measures may have mitigated the anticipated impact of crowding on virus transmission and mortality. This finding emphasizes the importance of governance, public health infrastructure, and community compliance in managing pandemics. This suggests that population density, which assumed more opportunity to spread coronavirus, does not account for differences in mortality rates [28].

Other factors that may play a significant role in affecting the COVID-19 mortality rate are the governments' response to create public health measures/policies to reduce the spread of COVID-19 as well as the testing and contact tracing systems of different countries. The strictness and competence of execution of public health interventions, such as lockdowns, social distancing, mask mandates, and travel restrictions, significantly impact COVID-19 transmission and mortality rates. Effective implementation and adherence to these measures can reduce the spread of the virus. Age distribution and the prevalence of comorbidities within a population influence the mortality rate. Older populations and those with higher rates of conditions such as obesity, diabetes, and cardiovascular diseases are at greater risk of severe outcomes from COVID-19 [29]. Demography, geography, language, social equity, and disease patterns in different countries may affect responses to the COVID-19 pandemic as a function of income level [30,31]. Lastly, cultural attitudes towards the pandemic and trust in the government and science can influence compliance with public health measures and vaccination coverage as well, thereby impacting the COVID-19 mortality rate.

This study has several limitations. First, we believed that COVID-19-related deaths in some LI and LMI countries were underreported. This is largely due to limited healthcare resources, including insufficient expenditure for facilities, as well as inadequate access to rapid testing and nucleic acid testing (NAT). Second, the analysis did not account for country-specific public health measures, cultural practices, or comorbidities, which could significantly impact outcomes. Third, the use of cross-sectional data limits causal interpretations. This finding suggests that analyzing individual GDP per capita does not fully elucidate unusual trends in COVID-19 mortality among LMI countries. A multifactorial approach should be considered for a comprehensive understanding of mortality trends. Lastly, we acknowledge that no adjustments were made for potential confounding factors in our analysis. Further analysis that accounts for confounding factors such as multivariable modeling could help explain the observed associations.

## Conclusions

This study reveals that COVID-19 mortality rates are influenced by complex, income-level-specific factors. While higher-income countries benefit from economic resources and vaccine coverage, lower-income countries face unique challenges linked to structural and contextual factors. The findings highlight the critical need for tailored public health strategies that address socioeconomic and healthcare disparities. Further research is essential to better understand these dynamics and inform equitable and effective global pandemic responses.

## Appendices

Appendix 1

**Table 1:** Overall raw data used in the study * is defined as income levels categorized based on the Atlas GNI per capita (current USD) for 2023 as follows: "Low-income" is defined as ≤1,135 USD, "Lower-middle-income" as 1,136-4,465 USD, "Upper-middle-income" as 4,466-13,845 USD, and "High-income" as >13,845 USD. An exception is made for Afghanistan and Qatar, where the income level range is based on the year 2022. # is defined as cumulative confirmed death cases as of December 29, 2023. Death counts may be underestimated due to underreporting. Percent vaccination for Qatar and the United Arab Emirates was >99%, so we recorded them as 99.0%. Recent values of GNI per capita, Atlas method (current USD) of most countries were recorded in 2023 except Afghanistan and Qatar which were recently recorded in 2022. Recent values of GNI per capita, PPP (current international USD) of most countries were recorded in 2023 except Afghanistan, Lebanon, and Qatar which were recently recorded in 2022. ISO: International Organization for Standardization; GDP: gross domestic product; GNI: gross national income; PPP: purchasing power parity; N/A: not available

Continents	Country names	ISO code	Income category (Atlas method)*	Population (n) in 2023	Population density (persons/km²) in 2023	Death cases (n) as of December 29, 2023^#^	Death cases /100k population (ratio) in 2023#	Percent vaccination (last updated March 13, 2023)	GDP (current USD) in 2023	GDP per capita (current USD) in 2023	GNI per capita, Atlas method (current USD) in 2023	GNI per capita, PPP (current international USD) in 2023	Infant mortality rate (deaths/1,000 live births) in 2023
Asia and Oceania	Afghanistan	AFG	Low-income	41,454,761	63.0	7,970	19.2	33.0	14,502.2	352.6	360.0	2,100.0	103.1
Africa	Algeria	DZA	Upper-middle-income	46,164,219	19.0	6,881	14.9	18.0	239,899.5	5,260.2	4,960.0	16,790.0	18.8
Africa	Angola	AGO	Lower-middle-income	36,749,906	29.0	1,937	5.3	48.0	84,723.0	2,309.5	2,130.0	7,310.0	57.2
North and South America	Argentina	ARG	Upper-middle-income	45,538,401	17.0	130,472	286.5	92.0	640,591.4	13,730.5	12,520.0	28,710.0	9.1
Asia and Oceania	Australia	AUS	High-income	26,451,124	3.0	24,273	91.8	88.0	1,723,827.2	64,711.8	63,140.0	66,260.0	3.0
Europe	Austria	AUT	High-income	9,130,429	111.0	22,534	246.8	78.0	516,034.1	56,506.0	55,070.0	73,520.0	3.2
North and South America	Bahamas	BHS	High-income	399,440	40.0	846	211.8	45.0	14,338.5	34,749.6	31,990.0	33,790.0	9.6
Asia and Oceania	Bangladesh	BGD	Lower-middle-income	171,466,990	1317.0	29,477	17.2	93.0	437,415.3	2,529.1	2,860.0	9,430.0	29.6
Europe	Belgium	BEL	High-income	11,712,893	387.0	34,339	293.2	81.0	632,216.6	53,475.3	54,530.0	71,990.0	3.2
North and South America	Bolivia	BOL	Lower-middle-income	12,244,159	11.0	22,385	182.8	64.0	45,849.8	3,701.0	3,600.0	10,490.0	22.3
Africa	Botswana	BWA	Upper-middle-income	2,480,244	4.0	2,800	112.9	84.0	19,395.8	7,249.8	7,620.0	19,140.0	24.4
North and South America	Brazil	BRA	Upper-middle-income	211,140,729	25.0	702,116	332.5	90.0	2,173,665.7	10,043.6	9,070.0	19,990.0	13.1
Europe	Bulgaria	BGR	High-income	6,795,803	63.0	38,619	568.3	30.0	101,584.4	15,797.6	14,460.0	37,380.0	7.8
Africa	Burkina Faso	BFA	Low-income	23,025,776	84.0	400	1.7	22.0	20,324.6	874.1	850.0	2,610.0	48.2
Asia and Oceania	Cambodia	KHM	Lower-middle-income	17,423,880	99.0	3,056	17.5	93.0	31,772.8	1,875.1	1,810.0	5,460.0	28.8
North and South America	Canada	CAN	High-income	39,299,105	4.0	54,307	138.2	92.0	2,140,085.6	53,371.7	53,930.0	60,700.0	4.3
Africa	Chad	TCD	Low-income	19,319,064	15.0	194	1.0	39.0	13,149.3	719.4	710.0	1,940.0	64.0
North and South America	Chile	CHL	High-income	19,658,835	26.0	62,203	316.4	95.0	335,533.3	17,093.2	15,820.0	31,590.0	6.4
Asia and Oceania	China	CHN	Upper-middle-income	1,422,584,933	152.0	121,889	8.6	94.0	17,794,782.0	12,614.1	13,400.0	24,380.0	6.5
North and South America	Colombia	COL	Upper-middle-income	52,321,152	47.0	142,727	272.8	85.0	363,540.2	6,979.7	6,870.0	21,420.0	11.7
North and South America	Costa Rica	CRI	High-income	5,105,525	100.0	9,366	183.4	91.0	86,497.9	16,595.4	13,850.0	25,800.0	7.0
Europe	Croatia	HRV	High-income	3,896,023	70.0	18,664	479.1	57.0	82,688.8	21,459.8	20,670.0	45,950.0	8.6
Africa	Democratic Republic of Congo	COD	Low-income	105,789,731	47.0	1,468	1.4	13.0	66,383.3	649.1	660.0	1,620.0	59.1
Europe	Denmark	DNK	High-income	5,948,136	140.0	9,418	158.3	82.0	404,198.8	67,967.4	73,360.0	79,390.0	3.0
North and South America	Dominica	DMA	Upper-middle-income	66,510	89.0	74	111.3	N/A	654.0	8,953.9	8,920.0	17,650.0	11.0
North and South America	Ecuador	ECU	Upper-middle-income	17,980,083	72.0	36,024	200.4	88.0	118,844.8	6,533.4	6,510.0	15,510.0	11.2
Africa	Egypt	EGY	Lower-middle-income	114,535,772	115.0	24,830	21.7	55.0	395,926.1	3,512.6	3,900.0	17,990.0	17.3
North and South America	El Salvador	SLV	Upper-middle-income	6,309,624	305.0	4,230	67.0	72.0	34,015.6	5,344.2	4,920.0	11,750.0	11.9
Europe	Estonia	EST	High-income	1,367,196	32.0	2,967	217.0	66.0	40,744.9	29,823.7	27,240.0	47,350.0	3.4
Africa	Ethiopia	ETH	Low-income	128,691,692	129.0	7,574	5.9	39.0	163,697.9	1,293.8	1,130.0	3,100.0	33.9
Europe	Finland	FIN	High-income	5,601,185	18.0	11,395	203.4	82.0	300,187.2	53,755.9	53,390.0	64,940.0	2.1
Europe	France	FRA	High-income	66,438,822	121.0	168,039	252.9	82.0	3,030,904.1	44,460.8	45,070.0	62,130.0	3.1
Africa	Gabon	GAB	Upper-middle-income	2,484,789	10.0	307	12.4	14.0	20,516.1	8,420.1	7,960.0	19,710.0	27.7
Europe	Germany	DEU	High-income	84,548,231	243.0	174,979	207.0	78.0	4,456,081.0	52,745.8	53,970.0	72,110.0	3.1
Africa	Ghana	GHA	Lower-middle-income	33,787,914	148.0	1,462	4.3	42.0	76,370.4	2,238.2	2,340.0	7,370.0	31.9
Europe	Greece	GRC	High-income	10,242,908	79.0	38,327	374.2	74.0	238,206.3	22,990.0	22,580.0	40,880.0	3.5
North and South America	Guatemala	GTM	Upper-middle-income	18,124,838	169.0	20,201	111.5	54.0	102,050.5	5,797.5	5,580.0	13,820.0	25.6
Africa	Guinea-Bissau	GNB	Low-income	2,153,339	77.0	177	8.2	28.0	1,966.5	914.3	900.0	2,670.0	47.7
Europe	Hungary	HUN	High-income	9,686,463	107.0	48,967	505.5	66.0	212,388.9	22,147.2	19,820.0	44,650.0	4.8
Asia and Oceania	India	IND	Lower-middle-income	1,438,069,596	484.0	533,333	37.1	75.0	3,549,918.9	2,484.8	2,540.0	10,030.0	30.4
Asia and Oceania	Indonesia	IDN	Upper-middle-income	281,190,067	155.0	161,954	57.6	75.0	1,371,171.2	4,940.5	4,870.0	15,210.0	19.3
Asia and Oceania	Iran	IRN	Upper-middle-income	90,608,707	56.0	146,751	162.0	79.0	401,504.5	4,502.5	4,680.0	17,900.0	14.6
Europe	Italy	ITA	High-income	59,499,453	202.0	195,179	328.0	84.0	2,254,851.2	38,373.2	38,200.0	58,650.0	3.1
Africa	Ivory Coast (Cote d'Ivoire)	CIV	Lower-middle-income	31,165,654	98.0	835	2.7	53.0	78,788.8	2,728.8	2,670.0	7,480.0	54
North and South America	Jamaica	JAM	Upper-middle-income	2,839,786	262.0	3,599	126.7	29.0	19,423.4	6,874.2	6,150.0	11,160.0	10.9
Asia and Oceania	Japan	JPN	High-income	124,370,947	341.0	74,694	60.1	83.0	4,212,945.2	33,834.4	39,030.0	52,640.0	1.9
Asia and Oceania	Jordan	JOR	Lower-middle-income	11,439,213	129.0	14,122	123.5	48.0	50,813.6	4,482.1	4,460.0	10,360.0	13.6
Asia and Oceania	Kazakhstan	KAZ	Upper-middle-income	20,330,104	8.0	19,072	93.8	59.0	261,421.1	13,136.6	10,940.0	35,420.0	8.1
Africa	Kenya	KEN	Lower-middle-income	55,339,003	97.0	5,689	10.3	27.0	107,440.6	1,949.9	2,110.0	6,220.0	26.9
Asia and Oceania	Lebanon	LBN	Lower-middle-income	5,773,493	564.0	10,947	189.6	40.0	17,937.3	3,350.3	3,740.0	12,490.0	6.9
Europe	Luxembourg	LUX	High-income	665,098	257.0	1,000	150.4	78.0	85,755.0	128,259.4	88,370.0	98,490.0	3.2
Africa	Madagascar	MDG	Low-income	31,195,932	54.0	1,426	4.6	8.6	16,031.7	528.7	530.0	1,840.0	38.3
Asia and Oceania	Malaysia	MYS	Upper-middle-income	35,126,298	107.0	37,268	106.1	88.0	399,648.8	11,648.7	11,970.0	36,120.0	6.5
Africa	Mali	MLI	Low-income	23,769,127	19.0	743	3.1	18.0	20,904.9	897.4	860.0	2,600.0	59.0
North and South America	Mexico	MEX	Upper-middle-income	129,739,759	67.0	334,126	257.5	78.0	1,788,886.8	13,926.1	12,100.0	24,970.0	12.0
Asia and Oceania	Mongolia	MNG	Upper-middle-income	3,431,932	2.0	2,136	62.2	70.0	19,872.2	5,764.8	4,950.0	16,390.0	19.8
Africa	Morocco	MAR	Lower-middle-income	37,712,505	85.0	16,298	43.2	69.0	141,109.4	3,672.1	3,700.0	9,600.0	18.7
Asia and Oceania	Nepal	NPL	Lower-middle-income	29,694,614	207.0	12,031	40.5	97.0	40,908.1	1,324.0	1,370.0	5,240.0	24.6
Europe	Netherlands	NLD	High-income	18,092,524	537.0	22,986	127.0	74.0	1,118,124.8	62,536.7	60,670.0	77,750.0	3.7
Asia and Oceania	New Zealand	NZL	High-income	5,172,836	20.0	3,623	70.0	87.0	253,465.7	48,527.8	48,610.0	52,750.0	3.4
North and South America	Nicaragua	NIC	Lower-middle-income	6,823,613	57.0	245	3.6	95.0	17,829.2	2,530.3	2,270.0	7,640.0	14.7
Africa	Nigeria	NGA	Lower-middle-income	227,882,945	250.0	3,155	1.4	39.0	362,815.0	1,621.1	1,930.0	6,200.0	55.2
Europe	Norway	NOR	High-income	5,519,167	15.0	5,732	103.9	81.0	485,513.3	87,961.8	102,460.0	108,790.0	1.8
Asia and Oceania	Pakistan	PAK	Lower-middle-income	247,504,495	321.0	30,656	12.4	75.0	338,368.5	1,407.0	1,500.0	6,110.0	52.7
North and South America	Panama	PAN	High-income	4,458,759	60.0	8,653	194.1	83.0	83,382.4	18,661.8	18,010.0	38,110.0	15.3
North and South America	Paraguay	PRY	Upper-middle-income	6,844,146	17.0	19,880	290.5	57.0	42,956.3	6,260.5	6,200.0	16,910.0	22.6
North and South America	Peru	PER	Upper-middle-income	33,845,617	26.0	220,516	651.5	93.0	267,603.3	7,789.9	6,990.0	15,860.0	10.8
Asia and Oceania	Philippines	PHL	Lower-middle-income	114,891,199	385.0	66,779	58.1	73.0	437,146.4	3,725.6	4,230.0	11,940.0	22.1
Europe	Poland	POL	High-income	38,762,844	127.0	120,159	310.0	60.0	811,229.1	22,112.9	19,730.0	47,380.0	5.1
Europe	Portugal	PRT	High-income	10,430,738	114.0	27,750	266.0	95.0	287,080.0	27,275.1	26,270.0	47,850.0	2.5
North and South America	Puerto Rico	PRI	High-income	3,242,023	366.0	5,938	183.2	N/A	117,902.3	36,779.1	25,240.0	32,990.0	5.9
Asia and Oceania	Qatar	QAT	High-income	2,979,082	257.0	690	23.2	99.0	235,770.4	87,480.4	70,070.0	116,870.0	6.5
Europe	Romania	ROU	High-income	19,118,479	83.0	68,690	359.3	42.0	351,002.6	18,419.4	16,670.0	46,620.0	5.7
Asia and Oceania	Saudi Arabia	SAU	High-income	33,264,292	15.0	9,646	29.0	79.0	1,067,582.9	28,895.0	28,690.0	55,290.0	12.0
Africa	Senegal	SEN	Lower-middle-income	18,077,573	94.0	1,971	10.9	16.0	31,014.0	1,746.0	1,660.0	4,670.0	31.8
Europe	Serbia	SRB	Upper-middle-income	6,773,201	77.0	18,057	266.6	48.0	75,187.1	11,361.0	10,030.0	25,880.0	4.6
Asia and Oceania	Singapore	SGP	High-income	5,789,090	8270.0	1,954	33.8	90.0	501,427.5	84,734.3	70,590.0	118,710.0	1.5
Europe	Slovakia (Slovak Republic)	SVK	High-income	5,518,055	115.0	21,201	384.2	48.0	132,793.6	24,470.2	22,790.0	43,500.0	5.3
Europe	Slovenia	SVN	High-income	2,118,396	105.0	9,487	447.8	61.0	68,216.8	32,163.5	30,620.0	54,130.0	1.5
Africa	South Africa	ZAF	Upper-middle-income	63,212,384	52.0	102,595	162.3	41.0	377,781.6	6,253.2	6,750.0	15,630.0	24.4
Europe	Spain	ESP	High-income	47,911,579	96.0	121,852	254.3	88.0	1,580,694.7	32,677.0	32,180.0	52,420.0	2.4
Africa	Sudan	SDN	Low-income	50,042,791	28.0	5,046	10.1	29.0	109,327.0	2,272.5	990.0	3,110.0	41.4
Europe	Switzerland	CHE	High-income	8,870,561	224.0	14,169	159.7	71.0	884,940.4	99,994.9	95,160.0	90,080.0	3.0
Africa	Tanzania	TZA	Lower-middle-income	66,617,606	75.0	846	1.3	58.0	79,158.3	1,211.1	1,210.0	3,900.0	30.3
Asia and Oceania	Thailand	THA	Upper-middle-income	71,702,435	140.0	34,514	48.1	82.0	514,945.0	7,171.8	7,180.0	22,880.0	6.4
Africa	Tunisia	TUN	Lower-middle-income	12,200,431	79.0	29,423	241.2	62.0	48,529.6	3,895.4	3,770.0	13,310.0	11.6
Asia and Oceania	Turkey (Türkiye)	TUR	Upper-middle-income	87,270,501	113.0	101,419	116.2	69.0	1,108,022.4	12,985.8	11,650.0	43,700.0	18.9
Africa	Uganda	UGA	Low-income	48,656,601	244.0	3,632	7.5	44.0	49,272.9	1,014.2	980.0	3,040.0	29.4
Europe	Ukraine	UKR	Upper-middle-income	37,732,836	65.0	109,918	291.3	36.0	178,757.0	5,181.4	5,070.0	18,560.0	9.0
Asia and Oceania	United Arab Emirates	ARE	High-income	10,642,081	127.0	2,349	22.1	99.0	504,173.5	52,976.8	53,290.0	83,750.0	5.1
Europe	United Kingdom of Great Britain	GBR	High-income	68,682,962	284.0	232,112	337.9	81.0	3,340,032.4	48,866.6	47,800.0	58,140.0	3.8
North and South America	United States of America	USA	High-income	343,477,335	38.0	1,162,244	338.4	80.0	27,360,935.0	81,695.2	80,300.0	82,190.0	5.1
North and South America	Uruguay	URY	High-income	3,388,081	19.0	7,625	225.1	87.0	77,240.8	22,564.5	19,530.0	31,690.0	8.1
Asia and Oceania	Vietnam	VNM	Lower-middle-income	100,352,192	324.0	43,206	43.1	94.0	429,717.0	4,346.8	4,180.0	14,400.0	14.4
Africa	Zimbabwe	ZWE	Lower-middle-income	16,340,822	42.0	5,731	35.1	44.0	26,538.3	1,592.4	1,740.0	3,850.0	34.0

Appendix 2

**Table 2:** Sources of data and their accessed dates ISO: International Organization for Standardization; GNI: gross national income; GDP: gross domestic product; PPP: purchasing power parity; N/A: not available

Topics	Online sources	Accessed date	Remarks
Continents	N/A	-	-
Country names	https://www.worldometers.info/	20-Sep-2024	-
ISO code	https://www.worldometers.info/	20-Sep-2024	-
Income category	N/A	-	Calculated based on Atlas GNI per capita (current USD)
Population (n) in 2023	https://www.worldometers.info/	19-Sep-2024	-
Population density (persons/km²) in 2023	https://www.worldometers.info/	19-Sep-2024	-
Death cases (n) as of December 29, 2023	https://ourworldindata.org/grapher/cumulative-deaths-and-cases-covid-19?tab=table&time=2020-01-08..2023-12-29	20-Sep-2024	-
Death cases /100k population (ratio) in 2023	N/A	-	Calculated from "Death case" divided by "Population density" and multiplied by 100,000
Percent vaccination (last updated March 13, 2023)	https://www.nytimes.com/interactive/2021/world/covid-vaccinations-tracker.html	19-Sep-2024	-
GDP (current USD) in 2023	https://data.worldbank.org/	14-Sep-2024	-
GDP per capita (current USD) in 2023	https://data.worldbank.org/	14-Sep-2024	-
GNI per capita, Atlas method (current USD) in 2023	https://data.worldbank.org/	14-Sep-2024	-
GNI per capita, PPP (current international USD) in 2023	https://data.worldbank.org/	14-Sep-2024	-
Infant mortality rate (deaths/1000 live births) in 2023	https://www.cia.gov/the-world-factbook/about/archives/2023/field/infant-mortality-rate/	17-Sep-2024	-

Appendix 3

**Table 3:** Descriptive and statistical analysis of socioeconomic factors * is defined as follows: groups sharing the same letter are not significantly different, while those with different letters are. GDP: gross domestic product; IMR: infant mortality rate

Topics	All countries (death cases /100k population (ratio))	High-income countries (death cases /100k population (ratio))	Upper-middle-income countries (death cases /100k population (ratio))	Lower-middle-income countries (death cases /100k population (ratio))	Low-income countries (death cases /100k population (ratio))	All countries (GDP per capita, USD)	High-income countries (GDP per capita, USD)	Upper-middle-income countries (GDP per capita, USD)	Lower-middle-income countries (GDP per capita, USD)	Low-income countries (GDP per capita, USD)	All countries (percent vaccination, %)	High-income countries (percent vaccination, %)	Upper-middle income countries (percent vaccination, %)	Lower-middle-income countries (percent vaccination, %)	Low-income countries (percent vaccination, %)	All countries (IMR, deaths/1,000 live births)	High-income countries (IMR, deaths/1,000 live births)	Upper-middle-income countries (IMR, deaths/1,000 live births)	Lower-middle-income countries (IMR, deaths/1,000 live births)	Low-income countries (IMR, deaths/1,000 live births)	All countries (population density, persons/km^2^)	High-income countries (population density, persons/km^2^)	Upper-middle-income countries (population density, persons/km^2^)	Lower-middle-income countries (population density, persons/km^2^)	Low-income countries (population density, persons/km^2^)
Number of values	95	38	25	22	10	95	38	25	22	10	93	37	24	22	10	95	38	25	22	10	95	38	25	22	10
Minimum	1.0	22.1	8.6	1.3	1.0	352.6	15,798.0	4,503.0	1,211.0	352.6	8.6	30.0	14.0	16.0	8.6	1.5	1.5	4.6	6.9	29.4	2.0	3.0	2.0	11.0	15.0
25% percentile	19.2	135.4	64.6	5.1	1.6	2,729.0	22,884.0	5,781.0	1,715.0	619.0	44.5	68.5	49.5	43.5	16.8	4.8	3.0	9.1	16.7	37.2	38.0	36.5	18.0	78.0	25.8
Median	112.9	214.4	116.2	19.6	5.3	8,420.0	37,576.0	7,172.0	2,507.0	885.8	73.0	81.0	73.5	60.0	28.5	11.2	3.6	11.9	27.9	48.0	89.0	106.0	65.0	107.0	58.5
75% percentile	252.9	330.5	269.7	46.9	8.7	32,164.0	58,014.0	11,505.0	3,679.0	1,084.0	84.5	87.5	87.3	79.5	39.0	25.6	6.0	19.6	32.4	60.3	155.0	228.8	126.5	321.8	95.3
Maximum	651.5	568.3	651.5	241.2	19.2	128,259.0	128,259.0	13,926.0	4,482.0	2,273.0	99.0	99.0	94.0	97.0	44.0	103.1	15.3	27.7	57.2	103.1	8,270.0	8,270.0	305.0	1,317.0	244.0
Range	650.5	546.2	642.9	239.9	18.2	127,907.0	112,462.0	9,424.0	3,271.0	1,920.0	90.4	69.0	80.0	81.0	35.4	101.6	13.8	23.1	50.3	73.7	8,268.0	8,267.0	303.0	1,306.0	229.0
Mean	149.9	233.0	168.9	50.1	6.3	21,299.0	45,971.0	8,349.0	2,647.0	951.6	65.2	76.7	66.5	61.8	27.4	17.8	4.8	14.4	28.6	52.4	221.5	347.8	82.2	227.7	76.0
Standard deviation	145.4	138.7	141.3	69.1	5.5	26,479.0	26,778.0	3,116.0	1,027.0	533.3	24.5	16.2	24.3	23.3	11.8	18.2	3.0	6.8	14.7	21.2	852.1	1,326.0	79.1	286.4	68.2
Standard error of the mean	14.9	22.5	28.3	14.7	1.7	2,717.0	4,344.0	623.3	218.9	168.6	2.5	2.7	5.0	5.0	3.7	1.9	0.5	1.4	3.1	6.7	87.4	215.1	15.8	61.1	21.6
Compact letter display (Kruskal-Wallis test: Dunn's multiple comparisons between groups for each factor using GraphPad Prism 10.2.1)*	-	A	A	B	B	-	A	B	C	C	-	A	A	A	B	-	A	AB	B	C	-	AB	A	B	AB
